# Supplementary material for: Relationship between Diet Quality and Socioeconomic and Health-Related Factors in Adolescents by Gender
Source: Nutrients. 2023 Dec 30;16(1):139. doi: 10.3390/nu16010139 (PMC10780370; doi:10.3390/nu16010139)
Supplement: Supplementary file 1 [file nutrients-16-00139-s001.zip › nutrients-2730267-Supplementary 1.pdf]

Supplementary Table S1

Criteria to define the score of each variable in the S-Healthy Eating Index					
**Variables**	**Criteria for maximum score of 10**	**Criteria for scoring 7.5**	**Criteria for scoring 5**	**Criteria for scoring 2.5**	**Criteria for the minimum score of 0**
Daily consumption					
1.Cereals and cereal products	Daily consumption	3 or more times a week but not daily	1 or 2 times a week	Less than once a week	Never or almost never
2.Vegetables	Daily consumption	3 o more times a week but not daily	1 or 2 times a week	Less than once a week	Never or almost never
3.Fruits	Daily consumption	3 or more times a week but not daily	1 or 2 times a week	Less than once a week	Never or almost never
4.Milk and dairy products	Daily consumption	3 or more times a week but not daily	1 or 2 times a week	Less than once a week	Never or almost never
Weekly consumption					
5.Meat	1 or 2 times a week	3 or more times a week but not daily	Less than once a week	Daily consumption	Never or almost never
6.Legumes	1 or 2 times a week	3 or more times a week but not daily	Less than once a week	Daily consumption	Never or almost never
Occasional consumption					
7.Processed meats	Never or almost never	Less than once a week	1 or 2 times a week	3 or more times a week but not daily	Daily consumption
8.Sweets	Never or almost never	Less than once a week	1 or 2 times a week	3 or more times a week but not daily	Daily consumption
9.Soft drinks	Never or almost never	Less than once a week	1 or 2 times a week	3 or more times a week but not daily	Daily consumption
10.Variety	2 points if meet each of the daily recommendations, 1 point if meet each of the weekly recommendations				

Source Norte-Navarro et al., 2011 [1]

1. Norte-Navarro, A.; Ortiz-Moncada, R. Calidad de la dieta española según el índice de alimentación saludable. *Nutr Hosp* **2011**, 330–336, doi:10.3305/nh.2011.26.2.4630.
