# Supplementary material for: Relationship between Diet Quality and Socioeconomic and Health-Related Factors in Adolescents by Gender
Source: Nutrients. 2023 Dec 30;16(1):139. doi: 10.3390/nu16010139 (PMC10780370; doi:10.3390/nu16010139)
Supplement: Supplementary file 1 [file nutrients-16-00139-s001.zip › nutrients-2730267-Supplementary 2.pdf]

**Supplementary Table S2: Distribution of frequencies of consumption of each food group according to Spanish Society of Community Nutrition dietary guidlines for boys and girls**

| | Percentage of reporting lower than recommended frequency of consumption | | Percentage of girls reporting recommended frequency of consumption | | Percentage of girls reporting higher than recommended frequency of consumption | |
|---|---|---|---|---|---|---|
| | Boys | Girls | Boys | Girls | Boys | Girls |
| **Cereals** | 81,6 | 88,0 | 16,6 | 11,27 | 1,4 | 0,7 |
| **Vegetables** | 91,2 | 88,2 | 8,8 | 11,8 | 0,0 | 0,0 |
| **Fruit** | 78,1 | 74,8 | 21,9 | 25,25 | 0,0 | 0,0 |
| **Milk and dairy products** | 70,6 | 74,8 | 29,4 | 25,25 | 0,0 | 0,0 |
| **Nuts** | 50,6 | 58,0 | 43,9 | 37,91 | 5,5 | 4,1 |
| **Legumes** | 38,1 | 39,6 | 60,0 | 59,26 | 1,9 | 1,1 |
| **Meat** | 8,2 | 15,4 | 24,7 | 30,86 | 67,2 | 53,6 |
| **Fish** | 47,5 | 47,3 | 32,4 | 33,85 | 20,1 | 18,9 |
| **Eggs** | 41,0 | 48,1 | 35,0 | 34,66 | 24,0 | 17,3 |
| **Processed meats** | 0,0 | 0,0 | 11,3 | 16,02 | 88,7 | 84,0 |
| **Soft drinks** | 0,0 | 0,0 | 28,1 | 47,64 | 72,0 | 52,4 |
| **Energy drinks** | 0,0 | 0,0 | 70,8 | 87,15 | 29,2 | 12,9 |
| **Sweets** | 0,0 | 0,0 | 51,7 | 49,29 | 48,3 | 50,7 |
| **Pastries** | 0,0 | 0,0 | 28,1 | 32,83 | 71,9 | 67,2 |
| **Snacks** | 0,0 | 0,0 | 29,9 | 35,06 | 70,1 | 64,9 |
| **Fast food** | 0,0 | 0,0 | 46,1 | 55,56 | 53,9 | 44,4 |
