# Supplementary material for: Relationship between Diet Quality and Socioeconomic and Health-Related Factors in Adolescents by Gender
Source: Nutrients. 2023 Dec 30;16(1):139. doi: 10.3390/nu16010139 (PMC10780370; doi:10.3390/nu16010139)
Supplement: Supplementary file 1 [file nutrients-16-00139-s001.zip › nutrients-2730267-supplementary 3.pdf]

**Supplementary Table S3: Sensitivity analysis using quantile regression models**

| | Boys S-HEI (n = 3505) | | | Girls S-HEI (n = 3814) | | |
|---|---|---|---|---|---|---|
| | Adjusted Coef. | 95% CI | *p* | Adjusted Coef. | 95% CI | *p* |
| **Course** | | | | | | |
| 2nd course of CSE | 0 | | | 0 | | |
| 4th course of CSE | −1.5 | (−2.4; −0.6) | **0.001** | 0.1 | (−0.8; 1.0) | 0.87 |
| 2nd course of PCSE | 1 | (-0.2; 2.2) | 0.096 | 2.3 | (1.3; 3.4) | **<0.001** |
| ILTC | −3.5 | (−5.2; −1.8) | **<0.001** | −3.5 | (−5.3; −1.6) | **<0.001** |
| **Parents' highest level of education** | | | | | | |
| University education | 0 | | | 0 | | |
| Secondary education | −2.0 | (−3.0; −1.0) | **<0.001** | −2.1 | (−3.0; −1.2) | **<0.001** |
| Primary education | −2.5 | (−3.7; −1.3) | **<0.001** | −3.8 | (−4.9; −2.7) | **<0.001** |
| No data | −3.5 | (−4.6; −2.4) | **<0.001** | −3.1 | (−4.2; −1.9) | **<0.001** |
| **Physical activity** [a] | | | | | | |
| Compliance with WHO recommendations | 0 | | | 0 | | |
| Under WHO recommendations | −0.5 | (−1.3; −0.3) | 0.238 | −1.0 | (−1.8; −0.2) | **0.019** |
| No data | −2.0 | (−3.6; −0.4) | **<0.001** | −3.5 | (−5.0; −1.1) | **<0.001** |
| **Alcohol use** [b] | | | | | | |
| Non-hazardous drinking | 0 | | | 0 | | |
| Hazardous drinking | −2.5 | (−3.9; −1.1) | **<0.001** | −0.6 | (−1.7; 0.5) | 0.31 |
| **Tobacco use** | | | | | | |
| Others | 0 | | | 0 | | |
| Daily use | −2.5 | (−4.2; −0.8) | **0.003** | −3.0 | (−4.4; −1.5) | **<0.001** |
| **Mobile use** [c] | | | | | | |
| No problematic use | 0 | | | 0 | | |
| Occasional or frequent problems | −2.0 | (−3.2; −0.8) | **0.001** | −1.5 | (−2.6; −0.3) | **0.011** |
| No data | −1.0 | (−2.0; −0.0) | **0.05** | −0.6 | (−1.6; 0.5) | 0.28 |
| **Self-perceived health** | | | | | | |
| Excellent/very good | 0 | | | 0 | | |
| Good | −1.5 | (−2.4; −0.6) | **0.001** | −1.5 | (−2.3; −0.7) | **<0.001** |
| Very poor/poor | −0.5 | (−2.1; 1.1) | 0.53 | −3.6 | (−4.9; −2.4) | **<0.001** |
| **Mood state** [d] | | | | | | |
| Good mood | | | | 0 | | |
| Low mood | | | | −1.3 | (−2.1; −0.4) | **0.006** |
| **Academic performance** | | | | | | |
| Good grades | 0 | | | 0 | | |
| Average grades | -1.5 | (−2.4; −0.6) | **0.001** | −2.0 | (−2.8;−1.1) | **<0.001** |
| Poor grades | −4.0 | (−5.4; −2.6) | **<0.001** | −5.6 | (−7.1; −4.0) | **<0.001** |
| No data | −3.5 | (−5.4; −1.6) | **<0.001** | −3.0 | (−4.9; −1.1) | **0.002** |

Statistically significant associations are highlighted in bold in the p-value columns. Abbreviations: 2nd and 4th course of CSE (Compulsory Secondary Education) corresponds to ISCED 2 (International Standard Classification of Education); 2nd PCSE (Post Compulsory Secondary Education) and ILTC (Intermediate Level Training Cycles) corresponds to ISCED3 (International Standard Classification of Education); WHO = World Health Organization; S-HEI = Healthy Eating Index Spanish adaptation; CI = Confidence Interval; "*p*" Student's *t* test, $p < 0.05$ were considered statistically significant. [a] In compliance with the WHO recommendation of ≥60 min per day, or under the WHO recommendation of 60 min per day, estimated from the average daily minutes of moderate or vigorous physical activity reported by the adolescents. [b] Scores above 3 on the Alcohol Use Disorders Identification Test (AUDIT-C test) were considered hazardous drinking. [c] Scores above 15 on the Questionnaire for mobile phone-related experiences (CERM test) were considered problematic mobile use. [d] Scores of 3 or more was identified as a low mood.
